# Perturbed gut microbiota and serum metabolites are associated with progressive renal fibrosis

**DOI:** 10.3389/fmed.2025.1489100

**Published:** 2025-04-28

**Authors:** Run-Xi Wang, Hong-Bing Zhou, Jia-Xing Gao, Xing-Hua Li, Wan-Fu Bai, Jia Wang, Ying-Chun Bai, Li-Ya Fan, Hong Chang, Song-Li Shi

**Affiliations:** ^1^Department of Pharmacy, Baotou Medical College, Inner Mongolia University of Science and Technology, Baotou, China; ^2^Institute of Bioactive Substance and Function of Chinese Materia Medica and Mongolian Medicine, Baotou Medical College, Inner Mongolia University of Science and Technology, Baotou, China; ^3^Changzhi People’s Hospital Affiliated to Changzhi Medical College, Changzhi, China

**Keywords:** chronic kidney disease, renal fibrosis, disease progression, oxidative stress, inflammation, biochemical markers

## Abstract

**Introduction:**

The intricate pathogenesis of renal fibrosis necessitates identifying biomarkers at various stages to facilitate targeted therapeutic interventions, which would enhance patient survival rates and significantly improve prognosis.

**Methods:**

We investigated the changes in gut microbiota and serum metabolites during the early, middle, and late stages of renal fibrosis in rats using 16S rDNA sequencing and UPLC-QTOF/MS-based metabolomics.

**Results:**

We identified 5, 21, and 14 potential gut microbial markers and 19, 23, and 31 potential metabolic markers in the MOD1, MOD2, and MOD4 groups, respectively. *Bifidobacterium* was identified as a shared microbial marker between the MOD1 and MOD2 groups; *Prevotellaceae_NK3B31_group* and *Bacteroides* were identified as shared microbial markers between the MOD2 and MOD4 groups. The pathways of arachidonic acid metabolism and retinol metabolism were found to play a significant role in the modulation of renal fibrosis at 1, 2, and 4 weeks. Notably, the metabolic biomarkers 8,9-EET and 5(S)-HPETE within these pathways emerged as critical determinants influencing renal fibrosis.

**Discussion:**

Our findings demonstrated that the severity of renal fibrosis is associated with dysbiosis of the gut microbiota and alterations in serum metabolites.

## Graphical Abstract

Mechanisms of renal fibrosis revealed through integrated gut microbiota and metabolomics analysis. 
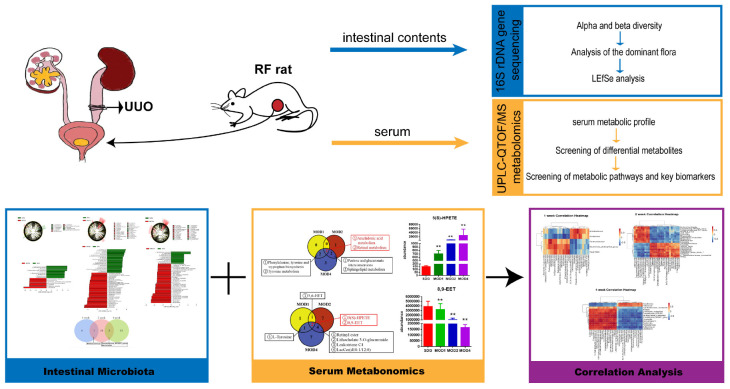

## Introduction

Renal fibrosis (RF) represents the ultimate common pathway of chronic kidney disease (CKD), encompassing diverse etiologies including glomerular or interstitial, congenital or acquired origins. Epidemiological studies indicate a global CKD prevalence of 10–14% (1). The global mortality rate of CKD continues to rise. In 2017, an estimated 1.2 million people died from CKD. By 2019, CKD had ranked 18th among global causes of death, and notably 8th among the elderly population aged 50–74 (2). Projections indicate that CKD will rise to the fifth leading cause of death worldwide by 2040 (3). Pathogenesis of RF is a multifaceted process characterized by excessive self-repair in the kidney following injury. Activated fibroblasts with low α-smooth muscle actin expression and myofibroblasts are key players in fibrosis (3). The initiation of RF involves intricate crosstalk between innate and adaptive immune systems, characterized by M1 macrophage-driven pro-inflammatory signals and M2 macrophage-mediated repair mechanisms. This chronic inflammatory milieu, a defining feature of CKD, constitutes a pivotal pathogenic trigger for fibrotic cascades (4). In the middle stage of RF, fibrosis ensues. The progression of RF involves a cascade of cellular events, including renal cell injury, inflammatory cell infiltration, myofibroblast activation, tubular atrophy, and microvascular rarefaction (5). Fibroblasts and perivascular pericytes are activated to proliferate, acquiring a myofibroblast phenotype through α-smooth muscle actin expression, which drives their transformation into the primary extracellular matrix-producing cells (6). In the early and middle stages of RF, disease reversal can be achieved through vasodilator, anti-inflammatory, and anticoagulant therapy. The renal fibrous tissue ultimately progresses to form irreversible scar tissue at the last of RF. However, safeguarding the integrity of healthy renal tissue is crucial in impeding the progression of RF, thereby extending patients’ lifespan and enhancing their quality of life (7). Consequently, it is imperative to ascertain the underlying factors contributing to RF and implement preventive measures or early interventions for effective management of CKD. The unilateral ureteral obstruction (UUO) of rodent model of has reached a high level of technical maturity, mirroring the developmental process observed in human RF. UUO induces renal hemodynamic and metabolic alterations, resulting in tubular injury and cellular apoptosis. It promotes infiltration of interstitial macrophages, subsequent differentiation into myofibroblasts, and their transformation into fibroblasts, ultimately leading to excessive extracellular matrix (ECM) deposition and establishment of a stable RF model (8).

Gut microbiota plays a pivotal role in modulating immune responses, maintaining metabolic homeostasis, and conferring resistance against pathogenic infections (9). CKD patients exhibited a significant enrichment of *Eggerthella lenta*, *Enterobacteriaceae*, *Clostridium* spp., and *Bacteroides fragilis*. Conversely, the abundance of *Bacteroides eggerthii*, *Roseburia faecis*, and *Prevotella* spp. decreased (10, 11). Alterations in the gut microbiota lead to the production of uremic toxic metabolites, precipitating oxidative stress, inflammation, renal impairment, and fibrosis, ultimately culminating in CKD (12). CKD can induce dysbiosis by altering the composition of intestinal bacteria, while the elevated levels of uremic toxins in individuals with CKD accelerate disease progression (13–15). Gut microbiota-derived metabolites serve as pivotal mediators of host-microbiome crosstalk, with dysbiosis-driven metabolite imbalance exacerbating renal inflammation and fibrosis, thereby accelerating CKD progression. Miao (16) revealed that selective modulation of *Lactobacillus johnsonii* attenuates RF and restores metabolic homeostasis, thereby offering a therapeutic avenue for CKD reversal. In Idiopathic Membranous Nephropathy patients, gut microbiota dysbiosis manifests as functional alterations, particularly driven by *Citrobacter*-mediated pro-inflammatory pathways and *Akkermansia*-associated tryptophan metabolism dysregulation, which collectively exacerbate renal injury (17). Continuous Ambulatory Peritoneal Dialysis induces gut microbial remodeling in end-stage renal disease (ESRD) patients, characterized by Firmicutes-predominated expansion of obligate anaerobic (*Blautia*), facultative anaerobic (*Lactobacillales*), and aerobic (*Bacilli*) lineages, reflecting adaptive responses to dialysis-associated metabolic stress (18). Our study is based on the concept of the “gut-kidney axis,” which elucidates the pathogenic interplay between intestinal microbiota and kidney diseases. The diverse components of intestinal microbiota, encompassing bacteria, viruses, protozoa, and fungi, exert profound influences on maintaining homeostasis as well as disease progression (19). The kidneys regulate plasma osmolality by precisely modulating the concentrations of water, solutes, and electrolytes in the bloodstream. Additionally, they possess distinct metabolic functions to uphold acid-base equilibrium and synthesize vital hormones.

Metabolomics is a comprehensive analytical approach that investigates small molecules (<1,500 Da) as metabolites in diverse biological samples, enabling the identification of metabolic alterations within biological systems that are intricately linked to disease pathogenesis. This field has gained widespread recognition for its indispensable role in disease diagnosis and evaluation, providing a fundamental basis for unraveling the mechanisms underlying various diseases (20, 21). Serum metabolomics can be employed to identify short-term biomarkers and provide a real-time reflection of the body’s current state, offering significant advantages in investigating clinical disease processes (22). The impact of RF on urinary metabolites in rats has been observed in studies (23), primarily affecting the synthesis of bioenergy by reducing the levels of branched chain amino acids and increasing the level of indole sulfate. The integration of intestinal microbiota and serum metabolomics offers a comprehensive and systematic approach to elucidate the pathogenesis of diseases, such as CKD (24), allergic rhinitis (25), and cardiometabolic disorders (26). Metabolomics has revealed that divergent renal diseases exhibit unique metabolic signatures, which not only distinguish pathological subtypes but also serve as potential biomarkers for targeted therapeutic interventions. Comparative metabolomics has revealed that divergent renal diseases exhibit unique metabolic signatures, which not only distinguish pathological subtypes but also serve as potential biomarkers for targeted therapeutic interventions (27). Wang et al. (28) discovered that exogenous adenine depletion induces renal dysfunction in rats, with phosphatidylcholines, lysophosphatidylcholines, and lysophosphatidic acids playing a predominant role, demonstrating lipid metabolism dysregulation as a critical contributor to CKD. Serum metabolomic analysis revealed severe dysregulation of purine, pyrimidine, and glutathione metabolic pathways in hyperuricemia, and identified key biomarkers including orotidine, ureidosuccinic acid, uracil, and pseudouridine as being associated with uric acid-induced hepatorenal injury (29). *Lactobacillus* ameliorates membranous nephropathy by modulating tryptophan-derived indole metabolites to inhibit the aryl hydrocarbon receptor pathway, with the reduced relative abundance of probiotics being positively correlated with decreased levels of indole-3-pyruvic acid, indole-3-aldehyde, and tryptamine, and negatively correlated with increased levels of indole-3-lactic acid and indole-3-acetic acid (30). The progression of diabetic nephropathy is associated with disorders of multiple metabolic pathways, including amino acid metabolism, arachidonic acid metabolism, pyrimidine metabolism, and the citrate cycle (31). Dahabiyeh et al. (32) compared serum samples between ESRD patients and CKD patients, identifying 193 significantly altered metabolites in ESRD. These metabolites were primarily involved in aminoacyl-tRNA biosynthesis, branched-chain amino acid biosynthesis, taurine metabolism, and tryptophan metabolism. We employed UPLC-QTOF/MS to investigate the metabolic profiles of rat serum and conducted high-throughput 16S rDNA sequencing of the gut microbiota. Differential bacteria and metabolic biomarkers were identified during 1, 2, and 4 weeks of RF treatment, enabling analysis of the alterations in three disease processes associated with RF. The alterations in the composition of gut microbiota and serum metabolites will provide us with comprehensive insights into the dynamics of gut flora and host metabolism. This valuable information will enhance our understanding of how RF impacts the dysbiosis of gut microbiota and subsequently influences the metabolic profiles in serum. Moreover, it will establish a novel theoretical foundation for identifying diagnostic biomarkers and potential therapeutic targets at different stages of RF.

## 2 Materials and methods

### 2.1 Chemicals and reagent

Pentobarbital sodium (solid, Merck KGaA, Darmstadt, Hesse, Germany); Penicillin sodium (solid, F7116323, North China Pharmaceutical Co., Ltd., Shijiazhuang, Heibei Privince, China); Non-absorbable surgical suture (Yangzhou Huanyu Medical Equipment Co., Ltd., Yangzhou, Jiangsu Province, China); Enzyme-linked immunosorbent assay (ELISA) kits for ALB, BUN, Scr, MDA, Col-III, Col-IV, HA, LN, IL-1β, IL-6, SOD, and HYP kits (Nanjing Jiancheng Technology Co., Ltd., Nanjing, Jiangsu Province, China); DNA extraction kit (DP712, Tiangen Biorech (Beijing) Co., Ltd., Beijing, China); Centrifuge (5424, Eppendorf AG, Hamburg, Germany); Vortex oscillator (WH0861, Hualida Scientific Instruments Co., Ltd., Changzhou, Jiangsu Province, China). ACQUITY HSS T3 column (2.1 × 100 mm, 1.8 μm; Waters Corporation, Milford, MA, United States). Methanol (Cat# A456-4, GC grade) and acetonitrile (Cat# A998-4, GC grade) were purchased from Thermo Fisher Scientific (Waltham, MA, United States); Liquid chromatography-tandem mass spectrometry (LC-MS/MS) analysis was performed using an ExionLC™ UPLC system coupled with a TripleTOF^®^ 5600+ high-resolution tandem mass spectrometer (SCIEX, Framingham, MA, United States).

### 2.2 Establishment of unilateral ureteral obstruction model

We purchased thirty-two healthy adult male SPF SD rats, weighing 170–200 g, from the Peking University Medical Department (Department of Experimental Animal Science, license number: SCXK [Beijing] 2022–0002). The room temperature was 22 ± 2°C, the relative humidity was 55–65%, and the light/dark cycle was 12 h. We divided the rats into a sham operation group (*n* = 8) and a model group (*n* = 24) after 1 week of adaptive feeding. The model group underwent UUO to establish a rat model of RF. The rats were anesthetized by intraperitoneal injection of 3% pentobarbital sodium at a dosage of 35 mg/kg, adjusted according to their body weight. The rats were positioned supine on a rat plate, and a longitudinal incision was made in the left lower abdomen to expose the left kidney layer by layer for identification of the ureter. The upper ureter was ligated with non-absorbable thread, except in the SDG group where it was separated without ligation.

### 2.3 Specimen collection

Samples were collected from the MOD group during the first, second, and fourth weeks, and from the SDG group during the fourth week. Rats were euthanized 24 h after the final administration using an intraperitoneal injection of 3% sodium pentobarbital. Following anesthesia, the rats were immobilized in a supine position, and a midline incision was made to access the abdominal cavity. Serum samples were collected from the abdominal aortic blood using centrifugation (Centrifuge operating parameters: 3500 r/20 min). The serum was subsequently stored at –80°C in a refrigerator for metabolomics research and the determination of biochemical indices. The cecum of the rats was promptly excised, and its contents were collected and preserved at –80°C for 16S rDNA sequencing. The intact ligated renal tissues of rats were collected and fixed with a 4% paraformaldehyde solution for hematoxylin-eosin (H&E) and Masson staining to observe the pathological changes in RF.

### 2.4 Determination of biochemical indicators and pathological analysis

The left kidney, fixed with a 4% poly-formaldehyde solution, was trimmed and placed in an embedding box. Following a 20-min flushing with running water, the kidney underwent two sequential elutions with gradient ethanol (80, 95, and 100%) for 15 min each to effectively remove residual water from the tissue. The tissues were placed in a preheated wax cylinder (in an incubator set at 70°C) for immersion in wax. The processed tissue was embedded in an embedding machine, and the wax blocks were subsequently frozen at –20°C on an ice table for a duration of 20 min. The wax blocks were extracted and sectioned into 3–4 μm slices for standard H&E and Masson staining. The serum levels of ALB, BUN, Scr, and tissue levels of SOD, MDA, HYP, IL-6, IL-1β, Col-III Col-IV LN, and HA were strictly detected following the instructions provided in the respective kits.

### 2.5 16S rDNA high-throughput sequencing

Samples of the gut contents from six rats in each experimental group were selected for analysis of the gut microbiota. The samples were subjected to DNA extraction using the CTAB method, and subsequent assessment of DNA quality was performed through agarose gel electrophoresis, followed by PCR amplification in a 25 μL reaction mixture. The universal primer set, 341F (5′-CCTACGGGNGGCWGCAG-3′) and 805R (5′-GACTACHVGGGTATCTAATCC-3′), was employed for amplification of the hypervariable V3-4 region within the 16S rDNA gene, targeting diverse bacterial taxa. PCR reaction conditions were performed as follows: 98°C 30 s; 98°C 10 s; 54°C 30 s, 35 cycles; 72°C 45 s; 72°C 10 min; 98°C 30 s. The PCR products were validated through electrophoresis on a 2% agarose gel.

The Agilent 2100 Bioanalyzer and Illumina Library Quantification Kit were used to assess the size and number of libraries. The NovaSeq PE250 platform was used to sequence the libraries and remove barcodes and primer sequences. FLASH software merged the paired readings. High-quality clean labels were obtained using fqtrim (version 0.94). The DADA2 algorithm was employed for length filtering and denoising, resulting in the generation of ASV (feature) sequences and corresponding abundance tables. The abundance of the samples was normalized, and alpha and beta diversity analyses were conducted based on characteristic sequences and abundance tables. The alpha diversity analysis evaluates diversity by six indices: observed_species, shannon, simpson, chao1, good_coverage, pielou_e. Beta diversity was mainly analyzed by calculating four distances (weighted_unifrac, unweighted_unifrac, jaccard, bray_curtis) using six analyses to assess the diversity between habitats (samples/groups). The species annotation was performed using the SILVA database (Release 138)^1^ according to the ASV (feature) sequence file, and the abundance of each species in each sample was counted according to the ASV (feature) abundance table. The confidence threshold for the annotation: 0.7. We identified the top 10 most abundant bacterial phyla and the top 30 most abundant bacterial genera for conducting differential analysis, aiming to investigate alterations in dominant bacteria during the RF process. Finally, LEfSe analysis (nsegata-lefse) was conducted with a threshold of LDA value > 3 and *p* < 0.05 to identify the bacterial taxa that exhibited significant differences across each week of RF treatment.

### 2.6 Metabolomic analysis

#### 2.6.1 Chromatographic and mass spectrometry conditions

The column temperature was 40°C; the flow rate was 0.4 mL/min; the injection volume was 1.0 μL; the mobile phase consisted of 0.1% (v/v) formic acid water in phase A and 0.1% (v/v) formic acid acetonitrile in phase B; the temperature of the autosampler was 4°C; gradient elution parameters: 0∼1.0 min, 1% B; 1.0∼3.5 min, 1%∼15% B; 3.5∼7.5 min, 15%∼25% B; 7.5∼9.0 min, 25%∼35% B; 9.0∼11.5 min, 35%∼99.9% B; 11.5∼17.0 min, 99.9% B; 17.0∼20.0 min, 99.9%∼1% B. A high-resolution tandem mass spectrometer Q-Exactive (Thermo Scientific) was used to detect metabolites eluted form the column. The Q-Exactive was operated in both positive and negative ion modes. Precursor spectra (70–1,050 m/z) were collected at 70,000 resolution to hit an AGC target of 3e6. The maximum inject time was set to 100 ms. A top 3 configuration to acquire data was set in DDA mode. Fragment spectra were collected at 17,500 resolution to hit an AGC target of 1e5 with a maximum inject time of 80 ms. Nitrogen was the dry gas and the temperature was set to 350°C. The cone hole’s voltage was 40.0 V. To ensure system stability and data reliability, we standardized the volume of all samples for composing quality control (QC) samples. We conducted QC testing prior to, during, and after sample injection.

#### 2.6.2 Metabolomics data processing

Chromatographic peak detection involves automated peak picking, alignment, and normalization using MassLynx (Waters) with the following parameters: a noise threshold of 1000, a minimum peak width of 5 s, and a mass error of less than 5 ppm. For missing value handling, peaks with more than 80% missingness across samples were removed, and the remaining missing values were imputed via k-nearest neighbors with k = 5. Principal component analysis (PCA) was used to assess global metabolic variation and identify outliers. Outliers were defined as samples outside the 95% confidence ellipse in score plots. Partial least squares discriminant analysis (PLS-DA) and orthogonal partial least squares discriminant analysis (OPLS-DA) were conducted in SIMCA-P 12.0 with unit variance scaling. The model validity was confirmed by permutation tests (*n* = 200, with R^2^Y and Q^2^ intercepts less than 0.4). Features with a VIP > 1.0 and an | log2FC| > 1.0 were prioritized. The volcano plot was generated to visualize the differential metabolites (upregulated or downregulated) across the comparisons of SDG *vs.* MOD1, MOD2, and MOD4. Level 1 annotation involves matching the retention time and MS/MS spectra to in-house standards. Level 2 annotation requires MS/MS spectral matching against public databases (HMDB)^2^ with a cosine similarity > 0.7, and endogenous substances were subsequently chosen.

### 2.7 Statistical analysis

Data analysis was performed using IBM SPSS Statistics (version 26.0) and GraphPad Prism (version 9.0). Continuous data are presented as median (interquartile range) to align with the non-parametric nature of the statistical tests. For comparisons between two independent groups with non-normally distributed data, the Mann-Whitney U test was applied. For comparisons across three or more groups, the Kruskal-Wallis test was employed, followed by Dunn’s post-hoc test for pairwise comparisons. A *p*-value < 0.05 was considered statistically significant. No correction for multiple comparisons was applied unless explicitly stated. All experiments included biological replicates (*n* ≥ 6 per group), defined as independent samples collected from distinct experimental units.

## 3 Results

### 3.1 Determination of biochemical indicators and histopathological analysis

**FIGURE 1:**
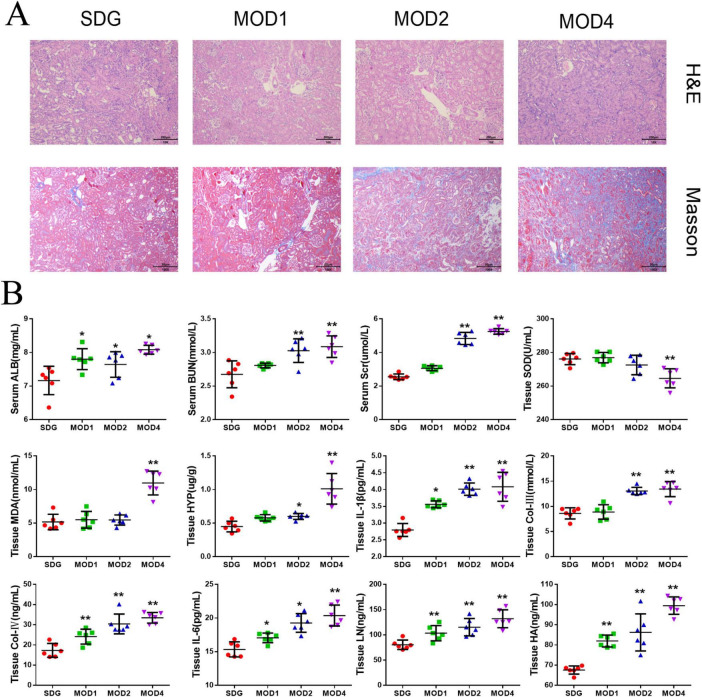
Biochemical indices and renal histopathological changes were evaluated in the SDG group as well as the MOD1, MOD2, and MOD4 groups. **(A)** The effect on histopathology of renal fibrosis rats (HE, Masson × 100). **(B)** Biochemical Index Results. Statistical analysis revealed significant differences (*p < 0.05 and **p < 0.01) compared to the SDG group.

The renal tissues of the sham operation (SDG) group and model groups (MOD1, MOD2, MOD4) were subjected to histological staining with HE and Masson’s trichrome. Subsequently, microscopic examination was performed to observe the histopathological alterations (Figure 1A). The HE staining revealed that, in comparison to the SDG group, the MOD1 group exhibited minimal infiltration of inflammatory cells, absence of prominent fibrotic structures, and relatively intact renal tissue architecture. The MOD2 group exhibited an increase in inflammatory cell infiltration, accompanied by tubular structural deformation and initiation of glomerular atrophy. The MOD4 group exhibited a significant presence of inflammatory cell infiltrations, focal fibrosis, tubular dilatation, as well as glomerular atrophy or even disappearance. Masson staining was employed to visualize collagen deposition in renal tissue, serving as an indicator of fibrosis severity. Masson staining revealed a significant increase in the area of collagen deposition with the progression of the RF model period. Biochemical parameters exhibited gradual changes in MOD1, MOD2, and MOD4 groups as depicted in Figure 1B. The levels of ALB, IL-6, and IL-1β (*p* < 0.05) exhibited significant increases in the MOD1, MOD2, and MOD4 groups. Moreover, Col-IV LN, and HA (*p* < 0.01) also demonstrated substantial elevation. The levels of ALB, IL-6, and IL-1β (*p* < 0.05) as well as Col-IVC LN, and HA (*p* < 0.01) exhibited significant increases in the MOD1, MOD2, and MOD4 groups. The levels of BUN, Scr, HYP, and Col-IV were significantly elevated in the MOD2 and MOD4 groups (*p* < 0.01; *p* < 0.05). Moreover, a significant increase in MDA level (*p* < 0.01) and a significant reduction in SOD activity (*p* < 0.01) were observed specifically in the MOD4 group.

### 3.2 Gut microbiota analysis of disease progression in renal fibrosis

#### 3.2.1 Evaluation of the diversity of gut microbiota in renal fibrosis rats

**FIGURE 2:**
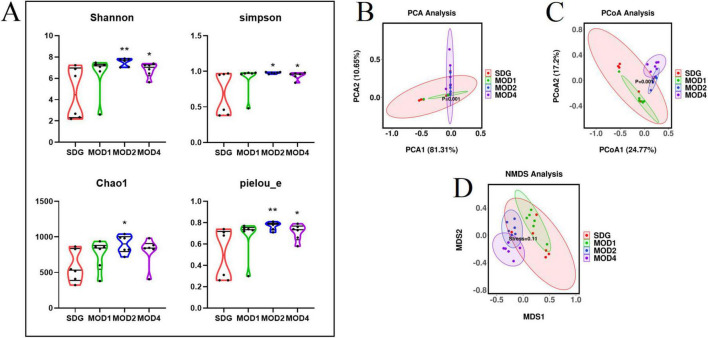
Analysis of bacterial diversity in RF. **(A)** Alpha diversity analysis, including Shannon index, Simpson index, Chao1 index and Pielou_e index. *p < 0.05 and **p < 0.01 *vs.* SDG group. **(B)** Beta diversity analysis was conducted within each group: **(C)** PCA; PCoA; **(D)** NMDS analysis.

The microbial composition of rat gut contents was analyzed using 16S rDNA gene sequencing, and the resulting characteristic sequence was obtained for diversity analysis, species annotation, and differential flora analysis. The alpha diversity indices, including Shannon, Simpson, Chao1, and Pielou_e indexes, were employed in our study to assess the species richness and evenness within the rat populations under investigation. Specifically, we compared these indices between the SDG group and each week of RF. The Shannon and Simpson indexes are indicative of species diversity and richness, respectively. The Chao1 index is primarily utilized for estimating the species abundance within a community. The Pielou_e index quantifies evenness, with higher values indicating greater uniformity. The alpha indexes of rats with RF exhibited varying degrees of increase on a weekly basis compared to the SDG group (Figure 2A). The Shannon, Simpson, Chao1, and Pielou_e indexes of the MOD2 group exhibited statistical significance (*p* < 0.01; *p* < 0.05). The Shannon, Simpson, and Pielou_e indexes of the MOD4 group were significant (*p* < 0.01; *p* < 0.05). Beta diversity encompasses the assessment of species diversity within and between environmental communities, enabling the detection of both intra- and inter-group variations. To evaluate the microbial composition and structure among the samples, PCA (Figure 2B) and principal co-ordinates analysis (PCoA) (Figure 2C) were performed on the differences of gut microbiota in each group. The results revealed a distinct segregation between the SDG group and the model groups, with each group exhibiting tight clustering. Compositional similarity among groups was evaluated using non-metric multidimensional scaling (NMDS) analysis (Figure 2D), which demonstrated varying degrees of separation between the MOD1, MOD2, and MOD4 groups compared to the SDG group. As RF progressed, the disparity between the model groups and the SDG group increased. The overall gut microbial composition differed significantly across all experimental groups.

#### 3.2.2 Analysis of differences in gut microbiota at the phylum and genus levels

**FIGURE 3:**
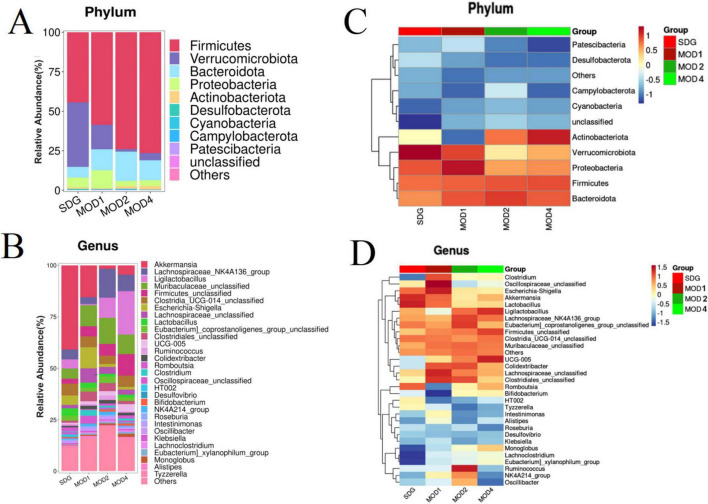
Heatmap of species composition and cluster analysis. **(A)** Stack map and **(C)** cluster heat map of top 10 at the phylum level. **(B)** Stack map and **(D)** cluster heat map of top 30 at the genus level. The degree of color saturation indicates the metabolite expression with red and blue, respectively indicating the highest and lowest expression.

**FIGURE 4:**
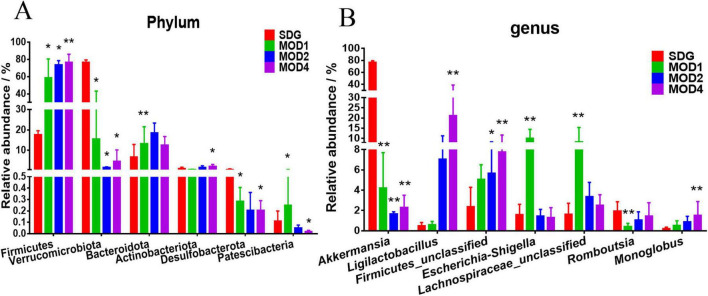
Analysis of significant differences in intestinal microbiota at the **(A)** phylum and **(B)** genus levels was conducted for each group. *p < 0.05 and **p < 0.01 *vs.* SDG group.

**FIGURE 5:**
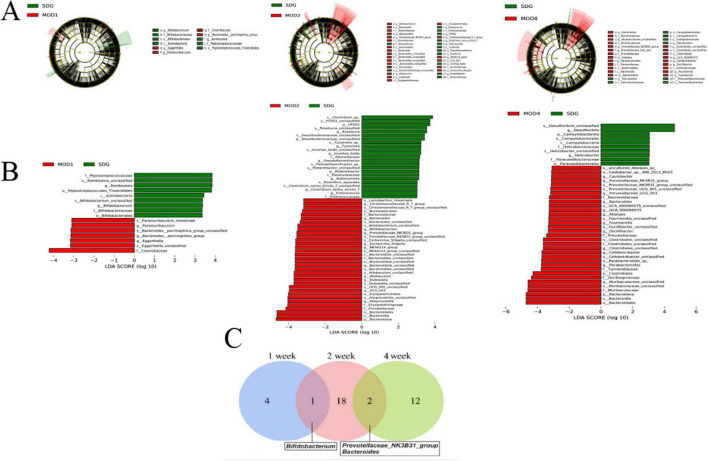
**(A)** LEfSe analysis and histogram of **(B)** LDA value distribution were performed on RF rats in each group. **(C)** A Venn plot was used to display the common differential bacterial biomarkers of RF at 1, 2, and 4 weeks (n = 6). LDA value > 3 and p < 0.05.

Based on the species abundance table and species annotation table, we selected the top 10 phyla with the highest abundance and the top 30 genera with the highest abundance for analysis of species composition and inter-sample clustering. The dominant bacterial phyla at the phylum level were Firmicutes, Verrucomicrobiota, Bacteroidota, Proteobacteria, Actinobacteriota, Desulfobacteria, Cyanobacteria, Campylobacteria, and Patescibacteria (Figure 3A). The dominant genera at the genus level were *Akkermansia*, *Lachnospiraceae_NK4A136_group*, *UCG-005*, *Ligilactobacillus*, *Muribaculaceae_unclassified*, *Firmicutes_ unclassified*, *Lactobacillus*, *Clostridia_UCG-014_unclassified*, *Clostridiales_unclassified*, *Lachnospiraceae_unclassified*, *Escher ichia-Shigella*, and *Ruminococcus* etc. (Figure 3B). The clustering heatmap depicts the similarity and dissimilarity of sample composition using a color gradient, where higher abundance is represented by shades of red and lower abundance is represented by shades of blue. In comparison to the SDG group, the dominant gut microbes exhibited altered abundance in the MOD1, MOD2, and MOD4 groups (Figures 3C,D). The significance of the disparity in gut microbial composition between the SDG group and the MOD groups was assessed at both phylum and genus levels (Figures 4A,B). In the MOD1 group, the relative abundance of Firmicutes, Bacteroidota, and Patescibacteria had significantly increased, while Verrucomicrobiota and Desulfobacteria had significantly decreased at the phylum level; the relative abundance of *Escherichia_Shigella* and *Lachnospiraceae_unclassified* had significantly increased, while *Akkermansia* and *Romboutsia* had significantly decreased at the genus level. In the MOD2 group, the relative abundance of Firmicutes had significantly increased, while Verrucomicrobiota had significantly decreased at the phylum level; the relative abundance of *Firmicutes_unclassified* had significantly increased, while *Akkermansia* had significantly decreased at the genus level. In the MOD4 group, the relative abundance of Firmicutes had significantly increased, while Verrucomicrobiota had significantly decreased at the phylum level; the relative abundance of *Escherichia_Shigella* and *Lachnospiraceae_unclassified* had significantly increased, while *Ligilactobacillus*, *Firmicutes_unclassified*, and *Monoglobushad* had significantly decreased at the genus level. LDA Effect Size (LEfSe) analysis was conducted to differentiate the variations in gut microbiota among RF rats at different stages (Figures 5A,B). According to LDA value > 3 and *p* < 0.05, microbiota with significant differences in abundance among different groups were selected. We observed differential microbiota at the genus level of RF. Five different markers were found in the MOD1 group: *Bifidobacterium* and *Romboutsia* were significantly lower, while *Eggerthella*, *Paramuribaculum*, and *Bacteroides_pectinophilus_group* were significantly higher. 21 different markers were found in the MOD2 group: *Incertae_Sedis*, *HT002*, *Roseburia*, *Desulfovibrionaceae_unclassified*, *Tyzzerella*, *Enterococcus*, *Pseudoflavonifractor*, *Rodentibacter*, *Robinsoniella*, and *Clostridium_sensu_stricto_7* were significantly lower, while *Bacteroides*, *Christensenellaceae_R_7_group*, *Escherichia_Shigella*, *Bifidobacterium*, *Allobaculum*, *UCG_005*, *Prevotellaceae_NK3B31_group*, *NK4A214_group*, *Dubosiella*, *Bacteroidota_unclassified*, and *Alloprevotella* were significantly higher. 14 different markers were found in the MOD4 group: *Desulfovibrio* and *Helicobacterwere* were significantly lower, while *Bifidobacterium*, *Bacteroides*, *Christensenellaceae_R_7_group*, *Allobaculum*, *Bacteroidota_unclassified*, *Dubosiella*, *UCG_005*, *Alloprevotella, Prevotellaceae_NK3B31_group*, *Escherichia_Shigella*, and *NK4A214*_*group* were significantly higher. *Bifidobacterium*, a common differential marker, underwent significant changes in the MOD1 and MOD2 groups; however, RF had different effects on it at the two stages. *Bacteroides* and *Prevotellaceae_NK3B31_group* were two common differential markers that changed significantly in the MOD2 and MOD4 groups, both of which exhibited upregulation (Figure 5C).

### 3.3 Serum metabolomics analysis of disease progression in renal fibrosis rats

#### 3.3.1 Changes in serum metabolic profile

**FIGURE 6:**
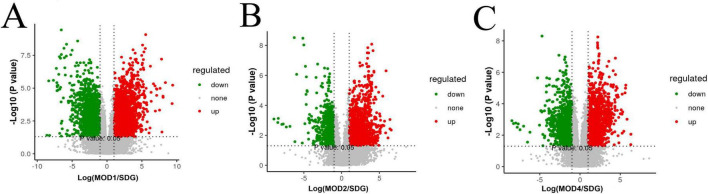
Metabolomics volcano plots of SDG *vs.* RF models at different stages (MOD1, MOD2, MOD4). **(A)** SDG *vs.* MOD1, **(B)** SDG *vs.* MOD2, **(C)** SDG *vs.* MOD4. Red/green dots indicate significantly up/downregulated metabolites (| log2FC| > 1.5, *p* < 0.05). Dashed lines mark thresholds.

**FIGURE 7:**
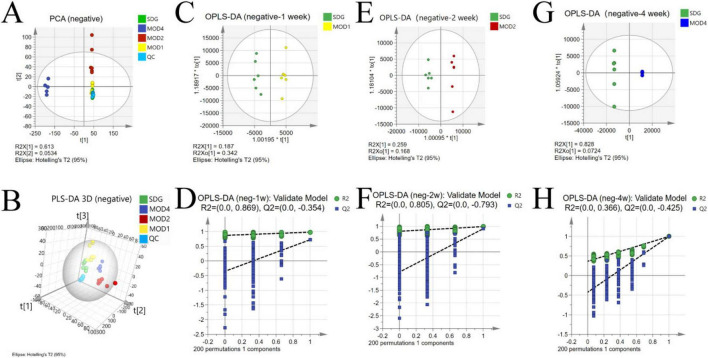
Alterations in the serum metabolic profile during RF under negative ion mode. PCA score plot **(A)**; 3D PLS-DA score plot **(B)** (R2X = 0.713, R2Y = 0.924, Q2 = 0.833); OPLS-DA score plot: MOD1 **(C)**, MOD2 **(E)**, MOD4 **(G)**; model validation: MOD1 **(D)**, MOD2 **(F)**, MOD4 **(H)**.

**FIGURE 8:**
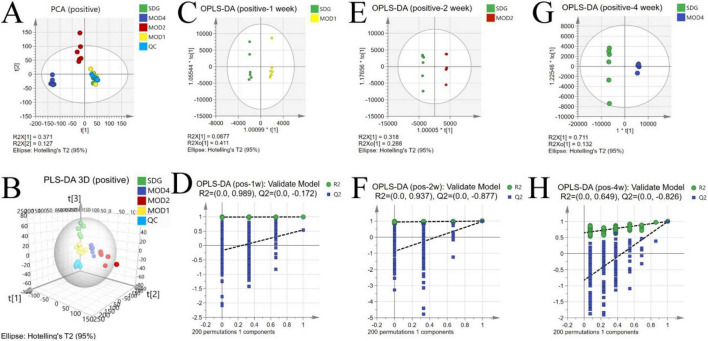
Alterations in the serum metabolic profile during RF under negative ion mode. PCA score plot **(A)**; 3D PLS-DA score plot **(B)** (R2X = 0.371, R2Y = 0.125, Q2 = 0.0522); OPLS-DA score plot: MOD1 **(C)**, MOD2 **(E)**, MOD4 **(G)**; model validation: MOD1 **(D)**, MOD2 **(F)**, MOD4 **(H)**.

The UPLC-QTOF/MS technique was employed for metabolomics analysis to investigate the alterations in serum metabolic profiles during RF. To ensure system stability, a QC assessment was conducted on every 10 samples throughout the injection process. We observed alterations in the serum metabolic profiles. The volcano plot (Figure 6), generated from univariate analysis comparing the control group and three distinct phases of renal fibrosis, visualizes metabolite changes across all detected compounds, including unannotated metabolites, with differential metabolites identified as those exhibiting a fold change (FC) > 1. In both positive and negative ion modes, the QC samples exhibited tight clustering in the PCA, confirming the stability and reproducibility of the method. While there was no clear separation between the MOD1 group and SDG group, the MOD2 and MOD4 groups showed distinct separation from the SDG group, demonstrating robust clustering within each respective group (Figures 7A, 8A). The PLS-DA and OPLS-DA are supervised statistical methods for discriminant analysis that effectively capture inter-group differences and enable accurate prediction of sample grouping. The similarity and dissimilarity of samples among two or more groups were assessed using PLS-DA analysis. Figures 7B, 8B depict the outcomes of the established 3D PLS-DA analysis in positive and negative ion modes, respectively. The distinctions among MOD2, MOD4, and SDG were apparent, while MOD1 exhibited some resemblance to the SDG group. OPLS-DA was employed to discriminate between the two sample groups, effectively highlighting their dissimilarities and elucidating potential biomarkers. The metabolites were analyzed in both positive and negative ion modes for each group (negative ion mode: Figures 7C,E,G; positive ion mode: Figures 8C,E,G). We observed a clear segregation between the MOD groups and the SDG group, and our permutation test (negative ion mode: Figures 7D,F,H; positive ion mode: Figures 8D,F,H) demonstrated that the model exhibited no signs of overfitting and displayed good predictive capability.

#### 3.3.2 Screening of differential metabolites

**FIGURE 9:**
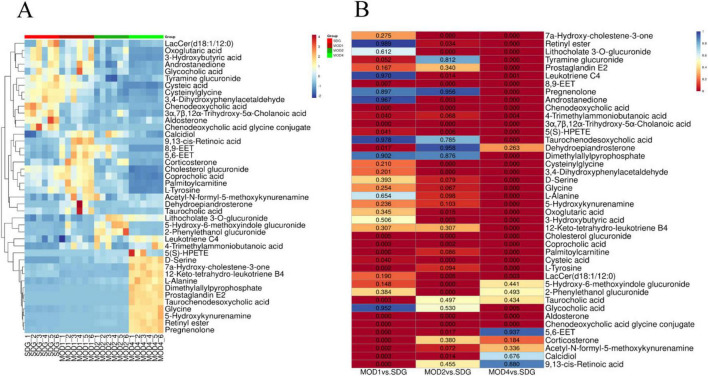
Differential metabolites abundance heatmap **(A)** and *p*-value heatmap **(B)** of RF.

Based on the results of OPLS-DA, the differential metabolites that met the requirements were further screened. Significant differential metabolites of RF at first, second, and fourth weeks with VIP > 1 and *p* < 0.05 were screened. Compared with the SDG group, there were 19 differential metabolites in the MOD1 group (Supplementary Table S1), 23 differential metabolites in the MOD2 group (Supplementary Table S2), and 31 differential metabolites in the MOD4 group (Supplementary Table S3). Cluster heatmap analysis was conducted to examine the abundance of differential metabolites involved in RF development (Figure 9A). The results revealed that a total of 42 metabolites were implicated in the regulation of RF progression, and these differential metabolites exhibited significant discriminatory power between the RF model groups and the SDG group. The *p*-value heatmap of differential metabolites in RF is depicted in Figure 9B. In the MOD1 group, 11 metabolites exhibited up-regulation, while eight showed down-regulation. In the MOD2 group, eight metabolites were found to be up-regulated, whereas 15 displayed down-regulation. In the MOD4 group, a total of 16 metabolites demonstrated up-regulation, while 15 exhibited down-regulation.

#### 3.3.3 Screening of metabolic pathways and critical biomarkers of renal fibrosis

**FIGURE 10:**
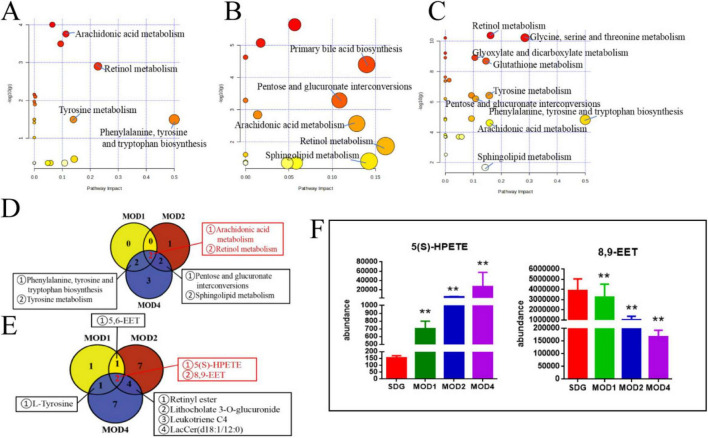
Critical metabolic pathways of RF: MOD1 **(A)**, MOD2 **(B)**, MOD4 **(C)**; Venn diagram of critical metabolic pathways **(D)**; Venn diagram of potential biomarkers in critical metabolic pathways **(E)**; co-regulated metabolites at 1, 2, and 4 weeks of RF **(F)**. **p < 0.01 *vs.* SDG group.

We conducted metabolomics pathway analysis^3^ on the differential metabolites and identified critical metabolic pathways based on Raw *p* > 0.05 and impact > 0.1 (Table 1). The MOD1 group exhibited four distinct metabolic pathways (Figure 10A): arachidonic acid metabolism, retinol metabolism, phenylalanine, tyrosine and tryptophan biosynthesis, and tyrosine metabolism. These pathways were found to involve five potential biomarkers. The MOD2 group exhibited the presence of five distinct metabolic pathways (Figure 10B): primary bile acid biosynthesis, pentose and glucuronate interconversions, arachidonic acid metabolism, retinol metabolism, and phingolipid metabolism. These pathways encompassed a total of 14 potential biomarkers. The MOD4 group exhibited the presence of nine metabolic pathways (Figure 10C): retinol metabolism, glycine, serine and threonine metabolism, glyoxylate and dicarboxylate metabolism, glutathione metabolism, tyrosine metabolism, pentose and glucuronate interconversions, phenylalanine, tyrosine and tryptophan biosynthesis, arachidonic acid metabolism, and sphingolipid metabolism. These pathways were found to involve a total of 16 potential biomarkers. Venn diagrams were utilized to visualize the intersection of critical metabolic pathways (Figure 10D) and metabolites (Figure 10E) at each stage of RF. There are two critical metabolic pathways that are jointly regulated at the first and second weeks, four critical metabolic pathways were co-regulated by the first and fourth weeks, and four critical metabolic pathways were co-regulated by the second and fourth weeks. Among them, the regulation of Arachidonic acid metabolism and Retinol metabolism is collectively governed by the first, second, and fourth weeks. Notably, two common metabolic biomarkers on these pathways were 8,9-EET and 5(S)-HPETE (Figure 10F). Importantly, these biomarkers exhibit distinct variations across the three stages of RF and hold significant potential as essential indicators influencing RF.

**TABLE 1:** Key metabolic pathways and metabolite in each week of RF.

Period	*N*	Pathway name	Hits	Raw *p*	Impact	Metabolite
1 week	1	Arachidonic acid metabolism	3	0.00017405	0.11317	5,6-EET; 5(S)-HPETE; 8,9-EET
	2	Retinol metabolism	1	0.0012547	0.22754	9,13-cis-Retinoic acid
	3	Phenylalanine, tyrosine and tryptophan biosynthesis	1	0.032166	0.5	L-Tyrosine
	4	Tyrosine metabolism	1	0.032166	0.13972	L-Tyrosine
2 weeks	1	Primary bile acid biosynthesis	7	0.000040132	0.13988	7a-Hydroxy-cholestene-3-one; Chenodeoxycholic acid; Chenodeoxycholic acid glycine conjugate; Glycocholic acid; Taurocholic acid; Taurochenodesoxycholic acid; Coprocholic acid
	2	Pentose and glucuronate interconversions	1	0.00051981	0.10843	Lithocholate 3-O-glucuronide
	3	Arachidonic acid metabolism	4	0.0027305	0.12845	Leukotriene C4; 8,9-EET; 5(S)-HPETE; 5,6-EET
	4	Retinol metabolism	1	0.013596	0.16168	Retinyl ester
	5	Sphingolipid metabolism	1	0.039965	0.14259	LacCer (d18:1/12:0)
4 weeks	1	Retinol metabolism	1	0.000000000042825	0.16168	Retinyl ester
	2	Glycine, serine and threonine metabolism	2	0.000000000060348	0.28464	Glycine; D-Serine
	3	Glyoxylate and dicarboxylate metabolism	1	0.0000000012372	0.10582	Glycine
	4	Glutathione metabolism	2	0.0000000020593	0.14553	Glycine; D-Serine
	5	Tyrosine metabolism	2	0.00000038475	0.15723	3,4-Dihydroxyphenylacetaldehyde; L-Tyrosine
	6	Pentose and glucuronate interconversions	1	0.00000062629	0.10843	Lithocholate 3-O-glucuronide; Cholesterol glucuronide; Tyramine glucuronide
	7	Phenylalanine, tyrosine and tryptophan biosynthesis	1	0.000015137	0.5	L-Tyrosine
	8	Arachidonic acid metabolism	5	0.000024437	0.15829	Prostaglandin E2; Leukotriene C4; 8,9-EET; 12-Keto-tetrahydro-leukotriene B4; 5(S)-HPETE
	9	Sphingolipid metabolism	1	0.020844	0.14259	LacCer (d18:1/12:0)

### 3.4 Correlation analysis between gut microbiota and metabolomic biomarkers during the process of renal fibrosis

**FIGURE 11:**
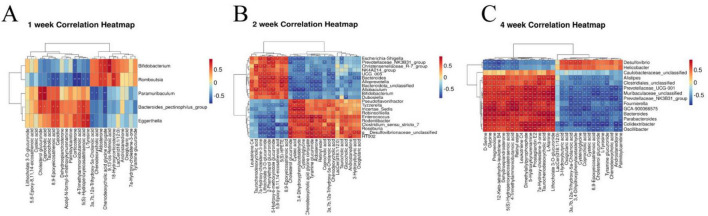
Heatmaps of correlation between intestinal microbiota and metabolomic biomarkers in rats with RF at the first week **(A)**, the second week **(B)**, and the fourth week **(C)**. *p < 0.05, **p < 0.01 *vs.* SDG group.

Spearman correlation analysis was conducted to examine the relationship between differential gut microbiota and key metabolites of RF. The color red indicates a positive correlation, while blue represents a negative correlation. The results of 1 week of RF (Figure 11A) showed significant positive correlations between 9,13-cis-retinoic acid and *Bifidobacterium*, L-Tyrosine and *Eggerthella*, as well as a negative correlation between L-Tyrosine and *Romboutsia* (*p* < 0.01, *p* < 0.05). The results of 2 weeks of RF (Figure 11B) showed significant negative correlations between *Incertae_Sedis* and taurochenodesoxycholic acid, while *Enterococcus* showed positive correlations with glycocholic acid, chenodeoxycholic acid glycine conjugate, and chenodeoxycholic acid, but a negative correlation with 7a-Hydroxy-cholestene-3-one. *HT002* exhibited positive associations with chenodeoxycholic acid glycine conjugate, chenodeoxycholic acid, coprocholic acid; however, it displayed a negative correlation with 7a-Hydroxy-cholestene-3-one. *Roseburia* and *Escherichia_Shigella* were negatively correlated with taurocholic acid and chenodeoxycholic acid but positively correlated with 7a-Hydroxy-cholestene-3-one. *Alloprevotella* was negatively associated with chenodeoxycholic acid glycine conjugate but positively correlated with 7a-Hydroxy-cholestene-3-one. *Christensenellaceae_R_7_group* and *Allobaculum* showed positive correlations with taurochenodesoxycholic acid and 7a-Hydroxy-cholestene-3-one. Additionally, *Allobaculum* had negative associations with chenodeoxycholic acid glycine conjugate, chenodeoxycholic aid, and glycoholcic acd; whereas *Escherichia_Shigella* exhibited positive relationships with taurohcolic acid, chendoeoxcyhlic acid, coprohclic acid, chenodeoxycholic acid glycine conjugate, but negative correlations with 7α-hydoxy-colestane-3-one (*p* < 0.01, *p* < 0.05). The results of 2 weeks of RF (Figure 11C) showed significant positive correlation between Glycine and D-Serine with *Alistipes*, *Caulobacter*, *Muribaculaceae_unclassified*, *Clostridiales_unclassified, Prevotellaceae_UCG_001, Fournierella, Colidextribacter*, and *Oscillibacter* (*p* < 0.01), while showing a negative correlation with *Desulfovibrio* and *Helicobacter* (*p* < 0.05).

## 4 Discussion

The pathogenesis of RF is multifaceted, involving dysbiosis of the gut microbiota and consequential alterations in host serum metabolism and inflammation, which collectively contribute to the development of RF. The UUO model shares etiological similarities with human obstructive nephropathy and serves as the most widely utilized rodent model for investigating non-immune-mediated tubulointerstitial fibrosis mechanisms. Through mechanical obstruction, the UUO model rapidly induces renal tubular epithelial cell injury, inflammatory infiltration, and fibrotic remodeling, effectively mimicking RF caused by urinary tract obstruction or tubulointerstitial pathologies in human chronic kidney disease (33). This study focuses on early-stage RF-associated metabolite alterations. The UUO model is particularly suited for dynamic metabolomic profiling due to its well-defined fibrotic progression and minimal confounding factors.

We employed UPLC-QTOF/MS metabolomics technology and 16S rDNA gut microbiota detection technology to identify the characteristic markers that impact disease progression. Our Study have observed the alterations in gut microbiota and serum metabolites across different stages of RF, demonstrating a significant correlation between them. RF is characterized by the accumulation of fibrotic tissue in the renal parenchyma, representing an excessive reparative response following kidney injury induced by various factors including trauma, infection, immune inflammation, and other insults. Fibrotic matrix deposition facilitates the reparative process during the early stages of RF progression and may undergo tissue remodeling following mild renal injury. Refractory fibrotic disease in its advanced stages is characterized by uncontrolled deposition of fibrotic matrix, structural damage to the kidneys, compromised blood supply, diminished organ function, and ultimately culminates in renal failure (34). In the pharmacodynamic analysis, the levels of serum ALB, BUN, Scr, and renal tissue SOD, MDA, HYP, (IL-6, IL-1β), Col-III, Col-IV, Colhe pharmacodynamic analysis, the levels of serum ALB, BUN, Scr, aent. SOD and MDA are indicative of oxidative reactions within the body (35, 36). The assessment of renal function commonly relies on the measurement of ALB, BUN, and Scr. ALB plays a crucial role in the inflammatory response (36), while BUN and Scr are metabolites that exhibit an inverse relationship with renal function levels (37). The increase in Scr levels primarily occurs during the middle and late stages of RF. Levels of HA, LN, Col-III The -IV Tand HYP are often positively correlated with the degree of fibrosis (38–41). At 1, 2, and 4 weeks of RF, significant increases were observed in levels of ALB, Col-IVo LN, HA, IL-6, and IL-1β (*p* < 0.01; *p* < 0.05). Additionally, at 2 and 4 weeks of RF, elevated levels were found for Col-IVo BUN, Scr, and HYP (*p* < 0.01; *p* < 0.05). However, the increase in MDA levels and decrease in SOD activity were observed at 4 weeks of RF progression (*p* < 0.01). The initial stages of RF exhibited alterations in cellular inflammation markers. Subsequent development of RF impaired renal excretory function, resulting in elevated levels of BUN and Scr, ultimately exacerbating RF through collagen fiber deposition and aggregation.

The gut microbiome metabolizes food components into bioavailable metabolites within the gut, and these resultant metabolites play a crucial role in regulating energy balance, nutrient uptake, and immune homeostasis (42–44). Zhou et al. (11) found that oral administration of live Bacillus fragilis alleviated renal fibrosis in UUO and adenine-induced mouse models and reduced lipopolysaccharide levels. Emerging evidence has established a strong association between alterations in the microbiome and its metabolome with the development of RF (10, 43, 45). The composition of the gut microbiota underwent two significant changes. Firstly, there was a dynamic enrichment or reduction in the microbiota throughout the entire early to late stage of RF. Secondly, the microbiota level changes only during specific period. Throughout the stages of RF development, we observed a significant increase in Firmicutes and a notable decrease in Verrucomicrobiota at the phylum level. Moreover, there was a marked reduction in the abundance of *Akkermansia* at the genus level. The predominant phylum, Firmicutes, is closely associated with the body’s immune response (46), and mice with RF exhibit an elevated relative abundance (47). In the UUO rats, the abundance of *Muribaculaceae_unclassified* and *UCG-005* was reduced (48, 49), which may lead to intestinal toxin accumulation, the occurrence or exacerbation of intestinal inflammation, and a decrease in SCFAs such as butyrate. Clinical studies have revealed that gut microbiota dysbiosis is associated with the production of uremic toxins. Gut microbiota disturbances (e.g., imbalance in the Firmicutes/Bacteroidetes ratio) exacerbate RF through the generation of uremic toxins such as indoxyl sulfate and p-cresyl sulfate. These toxins promote renal tubular epithelial inflammation and interstitial fibrosis by activating the NF-κB pathway and oxidative stress (50–52). CKD patients exhibit significant enrichment of toxin-producing *Clostridium* spp. and reduced abundance of butyrate-producing bacteria like Roseburia in the gut (10, 53). The gut microbiota member *Akkermansia*, belonging to the phylum Verrucomicrobia, is highly abundant in mammalian intestines. Its metabolites interact with and fortify the gut barrier, regulating metabolic and immune functions of both the gastrogut and circulatory systems. Additionally, these metabolites possess potential anti-inflammatory properties (54). Patients with inflammatory bowel disease and metabolic disorders exhibit decreased levels of *Akkermansia* (55). Pei et al. (56) demonstrated that administration of *Akkermansia* to 5/6 nephrectomy rats restored gut microecological disorders and reduced renal interstitial fibrosis, which is consistent with our findings. The initial stage of RF is typically asymptomatic, but it can progress to an irreversible state in its advanced stages, ultimately leading to renal failure. Therefore, the identification of biomarkers capable of distinguishing between early and late stages is crucial for timely intervention. At the 4 weeks, severe kidney damage occurs, leading to the production of numerous inflammatory reactions and fibrotic factors that facilitate an elevation in gut *monoglobus* level. *Monoglobus*, a pectin-degrading bacterium found in the human colon, has been shown to facilitate fibrosis reduction and enhance the absorption of blood ammonia through the portal vein system (57). Conversely, elevated levels of blood ammonia can lead to intestinal epithelial cell destruction and trigger inflammatory responses (58). LEfSe analysis identified 5, 21, and 14 markers of gut microbiota at 1, 2 and 4 weeks of RF, respectively. *Bifidobacterium* could be used as a common microbial marker at 1 and 2 weeks of RF, and *Prevotellaceae_NK3B31_group* and *Bacteroides* were used as common flora markers at 2 and 4 weeks of RF.

The investigation on acute kidney injury revealed a significant reduction in the abundance of *Bifidobacterium* (59). As a beneficial bacterium, *Bifidobacterium* can promote the production of short-chain fatty acids (SCFAs). SCFAs are produced through the bacterial breakdown of dietary fiber, and they exhibit histone deacetylase inhibitory properties that can impact renal physiological function and ameliorate renal injury (60, 61). However, it should be noted that *Prevotellaceae_NK3B31_group* and *Prevotellaceae_UCG_001* have been found to potentially induce intestinal inflammation and compromise intestinal health (62). The findings from microbiota research revealed a significant alteration in the gut microbiota as RF disease progressed. This alteration manifested as an increased diversity in bacterial abundance, with a dominance of detrimental bacteria within the gut.

Serum metabolomics can elucidate the intricate interactions between gut microbiota and distal organs and pathways (63, 64), providing valuable insights into the systemic effects of gut dysbiosis. It is also employed in the field of medicine to identify transient biomarkers that reflect the present physiological condition of the organism (22). Changes in metabolites such as aromatic hydrocarbons, indole, heterocyclic compounds, and fatty acids were influenced by RF induced by UUO (65). A total of 10 metabolic pathways were identified during the entire development of RF. Arachidonic acid metabolism and retinol metabolism are involved in the development of RF. 8,9-EET and 5(S)-HPETE are serum metabolic markers in these pathways. 8,9-EET is a regional isomer of Epoxyeicosatrienoic acids (EETs). EETs, synthesized by cytochrome P450 cyclooxygenase from arachidonic acid, exhibit various biological activities including vasodilation, inflammation reduction, anti-apoptosis, and inhibition of renal sodium reabsorption. These multifaceted effects contribute to the attenuation of blood pressure and deceleration of kidney disease progression (66). The lipid hydroperoxide precursor of leukotrienes is 5(S)-HPETE, which is derived from the reaction between 5-lipoxygenase and arachidonic acid. This process involves the formation of 5(S)-HPETE, followed by epoxide formation, ultimately resulting in leukotriene A4 from a single polyunsaturated fatty acid (67). Significant elevations in the level of 5(S)-HPETE diacylglycerol induce protein kinase activation, which subsequently triggers phospholipase A2 activation, leading to an augmented release of arachidonic acid. The inflammatory response is closely associated with the arachidonic acid metabolic pathway, and excessive levels of metabolites can contribute to renal inflammatory damage (53). Therefore, 8,9-EET and 5(S)-HPETE may serve as crucial foundations for RF diagnosis. We searched for metabolic biomarkers specific to the serum at each stage of RF. Pentose and glucuronate interchanges and Sphingolipid metabolism were common pathways for 2 and 4 weeks. The metabolic markers Lithocholate 3-O-glucuronide and LacCer (d18:1/12:0) were used to identify the severity of RF. Lithocholate 3-O-glucuronide is a metabolite of lithocholic acid in the body (68). Lithocholic acid glucuronide is found in the plasma and urine of people who have cholestatic syndrome. It is a bile inhibitor that harms hepatocytes and the body (69, 70). Studies have found that LacCer (d18:1/12:0) can cause metabolic disorders and oxidative stress (71, 72). The metabolic pathways of glycine, serine, and threonine metabolism, glyoxylate and dicarboxylate metabolism, as well as glutathione metabolism were found to be uniquely associated with the 4-week period. Notably, the metabolites glycine and D-serine can serve as discriminative markers for distinguishing between early and late stages of RF. The kidney, being a vital organ involved in the biosynthesis and catabolism of amino acids and their derivatives (73), plays a crucial role in maintaining renal tubular integrity. Additionally, glycine exhibits potent antioxidant properties that contribute to its renoprotective effects. Studies have demonstrated that (74) oral glycine administration in diabetic rats effectively mitigates kidney injury by primarily suppressing Nox4 mRNA and protein expression levels within the renal tissue. Additionally, D-amino acid oxidase plays a crucial role in regulating D-serine through its enzymatic breakdown of serine. The oxidative decomposition of D-serine generates H_2_O_2_, an intermediate product known for its high nephrotoxicity. Researchers (75) have observed significantly elevated serum levels of D-serine in mice with diabetic nephropathy, while the levels in their kidneys were notably lower. Given that D-serine is the most abundant amino acid in mammals, it can serve as a valuable biomarker for clinical diagnosis of CKD (76). Concurrently, microbiota-derived metabolites interact with host metabolism to influence RF progression. Abnormal metabolism of aromatic amino acids (e.g., tryptophan, phenylalanine) in CKD patients leads to the accumulation of breakdown products (e.g., indoxyl sulfate), which are negatively correlated with estimated glomerular filtration rate (eGFR) (24, 77). The relationship between amino acids and their derivatives and RF requires further investigation.

The human gut microbiota plays a crucial role in regulating diverse metabolic functions, encompassing enzymes, amino acid synthesis, bile acids (BAs) bioconversion, non-digestible carbohydrate fermentation, indole and polyamine production, as well as SCFAs. We analyzed and compared the changes in microbiota across different kidney disease models and the UUO model in previous studies. Kim observed that in an aged ischemia-reperfusion-induced kidney injury model, Prevotellaceae and Akkermansia were significantly increased compared to young mice, while Muribaculaceae and Bacteroidetes were significantly reduced (78). In chronic kidney disease accompanied by vascular calcification, the relative abundances of Asticcacaulis, Butyricicoccus, Desulfovibrio, Devosia, Lactobacillus, Lysobacter, and Prevotella were lower, but Adlercreutzia, Butyricimonas, Bacteroides, Turicibacter, Dehalobacterium, and Dorea showed significantly higher relative abundances (79). In diabetic kidney disease patients, dysregulated gut microbiota produced large amounts of LPS and gut-derived toxins, and disrupted bile acid metabolism, leading to an intestinal inflammatory environment and disruption of the intestinal epithelial barrier (80). To elucidate the gut microbiota associated with alterations in host serum metabolites, we analyzed correlations between key serum markers and the microbiota during the three periods of RF. At 1 week, 9,13-cis-retinoic acid was significantly positively correlated with *Bifidobacterium*. *Bifidobacterium* regulates the metabolism of SCFAs (59, 60) and the reduction of *Bifidobacterium* level at the early stage of RF has an impact on intestinal integrity, cell function and immunity. 9,13-cis-retinoic acid belongs to the retinoic acid (RA) subtype and is involved in cell growth, differentiation, apoptosis and inflammation (81, 82). Currently, more research is required to understand how RA affects fibrosis (83). Primary BAs were the main metabolites that changed during the 2 weeks. The liver synthesizes BAs from cholesterol, and intestinal microorganisms in the colon convert them into secondary BAs. BAs in the intestine have the capacity to modulate cholesterol, triglycerides, and fat-soluble vitamins digestion and absorption, while also playing a pivotal role in lipid and glucose metabolism (84). It has been found that (85) primary BAs biosynthesis is closely related to RF. Researchers discovered that *Christensenellaceae_R_7_group*, *Allobaculum*, and *Dubosiella* (86) influence the development of CKD. *Escherichia_Shigella* belonged to Proteobacteria, accounting for about 74%, and the increase in its level was a marker of lgA nephropathy (87). The pathogenesis of lgA nephropathy involves the stimulation of mesangial cell proliferation and subsequent release of a plethora of inflammatory mediators, resulting in elevated ALB levels. This cascade ultimately triggers RF and progresses to ESRD. We found that *Christensenellaceae_R_7_group*, *Allobaculum*, *Dubosiella*, and *Escherichia_Shigella* had positive effects on 2 weeks development of RF. While the abundance of beneficial bacteria associated with inflammation reduction, oxidative stress alleviation, organ fibrosis improvement, macrophage infiltration promotion, plasma endotoxin excretion enhancement, and gut microbiota balance was significantly reduced, including *Enterococcus* (88), *HT002* (89), and *Roseburia* (90). We observed the levels of glycine and D-serine significant increased at 4 weeks of RF, which were inversely correlated with the gut microbiota of *Desulfovibrio* and *Helicobacter*. It was positively correlated with *Caulobacter*, *Prevotellaceae_UCG_001*, *Muribaculaceae_unclassified*, *Alistipes*, *Oscillibacter*. *Caulobacter* has the ability to regulate proteolysis (91), which may contribute to its impact on the development of RF disease. The presence of Cytotoxin-associated gene A in *Helicobacter* enhances serum immunoglobulin A1 secretion and suppresses lgA1 glycosylation, potentially leading to lgA nephropathy (92). *Muribaculaceae_unclassified* and *Prevotellaceae_UCG_001* belong to Bacteroidetes, which can degrade polysaccharides and provide nutrients for other gut microbiota (93). Based on the correlation analysis of the three stages, we postulate that SCFAs and vitamin metabolism primarily influence the early stage of RF, while BAs play a significant role in the middle stage of RF, and amino acid metabolism becomes prominent during the late stage of RF.

The present study possesses certain limitations. Firstly, the sample size was relatively small. Considering the ethical implications of experimental research, animal studies were conducted with a carefully determined minimum sample size based on repeatability and representativeness. However, it remains challenging to completely eliminate the potential influence of environmental, dietary, and geographical factors on the progression of RF. Secondly, the pathogenesis and developmental processes of RF are intricate. UUO serves as a relatively expeditious and dependable RF model, effectively emulating clinical RF characteristics. However, the RF induced by UUO is limited to acute mechanical injury and may not encompass all manifestations of renal tubular and interstitial injury and fibrosis. Further investigation is warranted to explore RF caused by other factors such as drug abuse and bacterial infection. Finally, this study is grounded in the analysis of gut microbiota and serum metabolomics, providing a foundation for future investigations into the underlying mechanisms through which RF influences physiological biomarkers.

## 5 Conclusion

**FIGURE 12:**
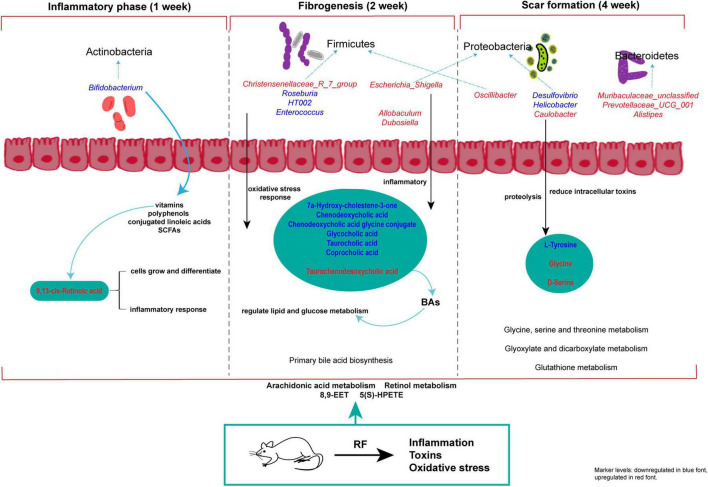
Study on the progression of RF disease based on metabolomics and intestinal flora. The red and blue characters in the figure indicate increased and decreased metabolite and intestinal flora abundance in each model group compared with the SDG group.

Metabolic pathways involved regulating of the whole stage of RF development include arachidonic acid metabolism and retinol metabolism, with 8,9-EET and 5(S)-HPETE as the major metabolic markers. At 1 week, metabolite 9, 13-cis-retinoic acid and *Bifidobacterium* are involved in the early development of RF. *Christensenellaceae_R_7_group*, *Allobaculum*, *Dubosiella* and other bacteria affected the further development of RF during 2 weeks. At 4 weeks, *Caulobacter*, *Helicobacter*, *Prevotellaceae_UCG_001*, etc. were involved in regulated amino acid metabolism. Glycine and D-serine were characteristic metabolic markers. In conclusion, our findings establish a significant association between intestinal microbiota and serum metabolites at distinct stages of RF (Figure 12), thereby providing a potential foundation for diagnosing and distinguishing the three disease stages of RF based on microbiome and metabolome profiles.

## Data Availability

The original contributions presented in the study are publicly available. All raw Illumina sequencing data have been deposited in the NCBI SRA under BioProject accession number [PRJNA1249965] (https://www.ncbi.nlm.nih.gov/sra/PRJNA1249965). Mass spectrometry data for the metabolomics metabolites are available in the Supplementary material.

## Footnotes

1
https://www.arb-silva.de/documentation/release138/

2
https://hmdb.ca/

3
https://www.metaboanalyst.ca/
