# Supplementary material for: Perturbed gut microbiota and serum metabolites are associated with progressive renal fibrosis
Source: Front Med (Lausanne). 2025 Apr 28;12:1489100. doi: 10.3389/fmed.2025.1489100 (PMC12068064; doi:10.3389/fmed.2025.1489100)
Supplement: Supplementary file 1 [file Table_1.docx]

Supplement 1. Identification of differential metabolites in rats with RF at 1 week (*p < 0.05 **p < 0.01)

| **NO.** | **Ion mode** | **Rt(min)** | **m/z** | **Formula** | **RankMS2**  **hmdb** | **Metabolite** | **Second order peak** | **VIP** | **Trend**  **(MOD1 vs. SDG)** | **KEGG ID** | **KEGG**  **Pathway** |
| --- | --- | --- | --- | --- | --- | --- | --- | --- | --- | --- | --- |
| 1 | - | 6.1414 | 319.2264362 | C20H32O3 | HMDB0002232 | 8,9-Epoxyeicosatrienoic acid (8,9-EET) | 151,163,165,275,301,319 | 1.10028 | ↑** | C14769 | Arachidonic acid metabolism |
| 2 | - | 4.98005 | 437.2884159 | C24H40O4 | HMDB0000518 | Chenodeoxycholic acid | 311,314,325,343,353,366,371,373,391 | 1.11693 | ↓** | C02528 | Primary bile acid biosynthesis |
| 3 | - | 4.594233333 | 335.2215555 | C7H15NO2 | HMDB0001161 | 4-Trimethylammoniobutanoic acid | 58,85,99,114,144 | 1.07781 | ↑* | C01181 | Lysine degradation |
| 4 | - | 4.122583333 | 448.3044179 | C26H43NO5 | HMDB0000637 | Chenodeoxycholic acid glycine conjugate | 448 | 2.1289 | ↓** | C05466 | Primary bile acid biosynthesis |
| 5 | - | 4.004566667 | 359.1885279 | C21H28O5 | HMDB0000037 | Aldosterone | 311,329,341,359 | 2.09868 | ↓** | C01780 | Steroid hormone biosynthesis |
| 6 | - | 3.879533333 | 453.2828898 | C24H40O5 | HMDB0000432 | 3α,7β,12α-Trihydroxy-5α-Cholanoic acid | 345,371,377,389,407 | 1.15873 | ↓** | C02373 | Steroid hormone biosynthesis |
| 7 | - | 3.797383333 | 335.221456 | C20H32O4 | HMDB0001193 | 5(S)-Hydroperoxyeicosatetraenoic acid  (5(S)-HPETE) | 89,115,259,273,303,317 | 1.19252 | ↑* | C05356 | Arachidonic acid metabolism |
| 8 | - | 3.450733333 | 333.2057236 | C19H28O2 | HMDB0000077 | Dehydroepiandrosterone | 215,227,253,255,287 | 1.13621 | ↓* | C01227 | Steroid hormone biosynthesis |
| 9 | - | 3.218533333 | 391.2104192 | C21H30O4 | HMDB0001547 | Corticosterone | 269,287,301,309,327,329 | 2.39747 | ↓** | C02140 | Steroid hormone biosynthesis |
| 10 | - | 3.018366667 | 514.2833015 | C26H45NO7S | HMDB0000036 | Taurocholic acid | 514 | 1.51739 | ↑** | C05122 | Primary bile acid biosynthesis; Taurine and hypotaurine metabolism |
| 11 | - | 1.342466667 | 309.1087826 | C13H16N2O4 | HMDB0004259 | Acetyl-N-formyl-5-methoxykynurenamine | 146,161,177 | 2.2 | ↑** | C05642 | Tryptophan metabolism |
| 12 | + | 10.70118333 | 1147.817094 | C33H54O7 | HMDB0010330 | Cholesterol glucuronide | 111,273,327,369,563 | 2.089 | ↓** | C03033 | Pentose and glucuronate interconversions; Ascorbate and aldarate metabolism |
| 13 | + | 10.68688333 | 923.6627704 | C27H46O5 | HMDB0000601 | Coprocholic acid | 317,330,331,369,421,451 | 2.4493 | ↑** | C04722 | Primary bile acid biosynthesis |
| 14 | + | 10.33033333 | 801.6791853 | C27H44O2 | HMDB0003550 | Calcidiol | 69,273,289,313,327,341,385 | 6.79972 | ↑** | C01561 | Steroid biosynthesis |
| 15 | + | 6.156083333 | 343.2224295 | C20H32O3 | HMDB0002190 | 5,6-Epoxy-8,11,14-eicosatrienoic acid  (5,6-EET) | 85,99,201,205,233,273,285,303 | 4.15203 | ↑** | C14768 | Arachidonic acid metabolism |
| 16 | + | 5.543016667 | 400.3404595 | C23H45NO4 | HMDB0000222 | Palmitoylcarnitine | 341, 400 | 4.83757 | ↑** | C02990 | Fatty acid degradation |
| 17 | + | 5.489433333 | 301.2162571 | C20H28O2 | HMDB0012874 | 9,13-cis-Retinoic acid | 145,201,215,255,283 | 2.67131 | ↑** | C00777 | Retinol metabolism |
| 18 | + | 2.556316667 | 339.0172494 | C3H7NO5S | HMDB0002757 | Cysteic acid/  Cysteate | 105,124,137,170 | 1.77255 | ↓* | C00506 | Taurine and hypotaurine metabolism; Cysteine and methionine metabolism |
| 19 | + | 1.125766667 | 182.0806595 | C9H11NO3 | HMDB0000158 | L-Tyrosine | 94,106 | 1.76917 | ↑** | C00082 | Phenylalanine, tyrosine and tryptophan biosynthesis; Tyrosine metabolism; Ubiquinone and other terpenoid-quinone biosynthesis; Phenylalanine metabolism; |

Supplement 2. Identification of differential metabolites in rats with RF at 2 weeks (**p* < 0.05 ***p* < 0.01)

| **NO.** | **Ion mode** | **Rt(min)** | **m/z** | **Formula** | **RankMS2**  **hmdb** | **Metabolite** | **Second order peak** | **VIP** | **Trend**  **(MOD2 vs. SDG)** | **KEGG ID** | **KEGG**  **Pathway** |
| --- | --- | --- | --- | --- | --- | --- | --- | --- | --- | --- | --- |
| 1 | - | 10.42908333 | 445.3324263 | C27H44O2 | HMDB0001993 | 7a-Hydroxy-cholestene-3-one | 113,193,207,313,341,355,367,383 | 2.26514 | ↓** | C05455 | Primary bile acid biosynthesis |
| 2 | - | 9.8717 | 649.4403306 | C20H30O2 | HMDB0003598 | Retinyl ester | 241,255,283 | 1.87446 | ↑* | C02075 | Retinol metabolism |
| 3 | - | 8.052916667 | 1149.658174 | C30H48O9 | HMDB0002513 | Lithocholate 3-O-glucuronide | 117,163,175,357,375,433,463,551 | 2.28372 | ↑** | C03033 | Pentose and glucuronate interconversions |
| 4 | - | 7.94575 | 625.2308293 | C14H19NO7 | HMDB0010328 | Tyramine glucuronide | 117,118,175,283 | 2.28927 | ↓** | C03033 | Pentose and glucuronate interconversions |
| 5 | - | 6.866683333 | 624.2967309 | C30H47N3O9S | HMDB0001198 | Leukotriene C4 | 624 | 1.6353 | ↑* | C02166 | Arachidonic acid metabolism |
| 6 | - | 6.1414 | 319.2264362 | C20H32O3 | HMDB0002232 | 8,9-Epoxyeicosatrienoic acid  (8,9-EET) | 151,163,165,275,301,319 | 2.1425 | ↑** | C14769 | Arachidonic acid metabolism |
| 7 | - | 5.083766667 | 333.2060696 | C19H28O2 | HMDB0000899 | Androstanedione | 83,100,145,151,161,163,165,169,175,177,215,217,223,225,227, 240,25,265,269,285 | 2.00224 | ↓** | C00674 | Steroid hormone biosynthesis |
| 8 | - | 4.98005 | 437.2884159 | C24H40O4 | HMDB0000518 | Chenodeoxycholic acid | 311,314,325,343,353,366,371,373,391 | 2.34168 | ↓** | C02528 | Primary bile acid biosynthesis |
| 9 | - | 4.122583333 | 448.3044179 | C26H43NO5 | HMDB0000637 | Chenodeoxycholic acid glycine conjugate | 448 | 2.1289 | ↓** | C05466 | Primary bile acid biosynthesis |
| 10 | - | 4.004566667 | 359.1885279 | C21H28O5 | HMDB0000037 | Aldosterone | 311,329,341,359 | 2.09868 | ↓** | C01780 | Steroid hormone biosynthesis; |
| 11 | - | 3.879533333 | 453.2828898 | C24H40O5 | HMDB0000432 | 3α,7β,12α-Trihydroxy-5α-Cholanoic acid | 345,371,377,389,407 | 2.14223 | ↓** | C02373 | Steroid hormone biosynthesis |
| 12 | - | 3.072083333 | 355.0716826 | C5H10N2O3S | HMDB0000078 | Cysteinylglycine | 97,99,100,102,113,117,126,131,133,177 | 2.49573 | ↓** | C01419 | Glutathione metabolism |
| 13 | - | 2.98265 | 197.0443 | C8H8O3 | HMDB0003791 | 3,4-Dihydroxyphenylacetaldehyde | 121,123,133,135,151 | 2.44115 | ↓** | C04043 | Tyrosine metabolism |
| 14 | - | 2.43955 | 297.1008497 | C14H18O7 | HMDB0010350 | 2-Phenylethanol glucuronide | 89,117,163,175,279,297 | 2.33923 | ↑** | C03033 | Pentose and glucuronate interconversions; Ascorbate and aldarate metabolism |
| 15 | - | 1.864166667 | 338.0870965 | C15H17NO8 | HMDB0010363 | 5-Hydroxy-6-methoxyindole glucuronide | 89,117,146,147,163,175,308,338 | 2.34939 | ↑** | C03033 | Pentose and glucuronate interconversions |
| 16 | - | 1.17815 | 291.039057 | C5H6O5 | HMDB0000208 | Oxoglutaric acid | 73,101,144 | 1.7577 | ↓* | C00026 | Butanoate metabolism;Citrate cycle (TCA cycle); Alanine, aspartate and glutamate metabolism; Arginine biosynthesis; Lipoic acid metabolism |
| 17 | - | 1.174566667 | 103.0389122 | C4H8O3 | HMDB0000011 | 3-Hydroxybutyric acid | 59,102 | 1.95596 | ↓** | C01089 | Butanoate metabolism |
| 18 | - | 3.797383333 | 335.221456 | C20H32O4 | HMDB0001193 | 5(S)-Hydroperoxyeicosatetraenoic acid  (5(S)-HPETE) | 89,115,259,273,303,317 | 2.28112 | ↑** | C05356 | Arachidonic acid metabolism |
| 19 | + | 10.70118333 | 1147.817094 | C33H54O7 | HMDB0010330 | Cholesterol glucuronide | 111,273,327,369,563 | 1.93227 | ↓* | C03033 | Pentose and glucuronate interconversions |
| 20 | + | 10.68688333 | 923.6627704 | C27H46O5 | HMDB0000601 | Coprocholic acid | 317,330,331,369,421,451 | 1.2618 | ↓** | C04722 | Primary bile acid biosynthesis |
| 21 | + | 9.884466667 | 828.5483613 | C42H79NO13 | HMDB0004866 | LacCer(d18:1/12:0) | 628,788 | 5.06864 | ↓** | C01290 | Sphingolipid metabolism |
| 22 | + | 6.156083333 | 343.2224295 | C20H32O3 | HMDB0002190 | 5,6-Epoxy-8,11,14-eicosatrienoic acid  (5,6-EET) | 85,99,201,205,233,273,285,303 | 1.35081 | ↑* | C14768 | Arachidonic acid metabolism |
| 23 | + | 2.556316667 | 339.0172494 | C3H7NO5S | HMDB0002757 | Cysteic acid | 105,124,137,170 | 2.6782 | ↓** | C00506 | Cysteine and methionine metabolism; Taurine and hypotaurine metabolism |

Supplement 3. Identification of differential metabolites in rats with RF at 4 week (**p* < 0.05 ***p* < 0.01)

| **NO.** | **Ion mode** | **Rt(min)** | **m/z** | **Formula** | **RankMS2**  **hmdb** | **Metabolite** | **Second order peak** | **VIP** | **Trend**  **(MOD2 vs. SDG)** | **KEGG ID** | **KEGG**  **Pathway** |
| --- | --- | --- | --- | --- | --- | --- | --- | --- | --- | --- | --- |
| 1 | - | 10.42908333 | 445.3324263 | C27H44O2 | HMDB0001993 | 7a-Hydroxy-cholestene-3-one | 113,193,207,313,341,355,367,383 | 1.18229 | ↑** | C05455 | Primary bile acid biosynthesis |
| 2 | - | 9.8717 | 649.4403306 | C20H30O2 | HMDB0003598 | Retinyl ester | 241,255,283 | 1.20776 | ↑** | C02075 | Retinol metabolism |
| 3 | - | 8.052916667 | 1149.658174 | C30H48O9 | HMDB0002513 | Lithocholate 3-O-glucuronide | 117,163,175,357,375,433,463,551 | 1.15734 | ↑** | C03033 | Pentose and glucuronate interconversions |
| 4 | - | 7.94575 | 625.2308293 | C14H19NO7 | HMDB0010328 | Tyramine glucuronide | 117,118,175,283 | 1.07892 | ↓** | C03033 | Pentose and glucuronate interconversions |
| 5 | - | 7.8243 | 397.2256423 | C20H32O5 | HMDB0001220 | Prostaglandin E2 | 175,270,316,351 | 1.20698 | ↑** | C00584 | Arachidonic acid metabolism |
| 6 | - | 6.866683333 | 624.2967309 | C30H47N3O9S | HMDB0001198 | Leukotriene C4 | 624 | 1.10781 | ↑** | C02166 | Arachidonic acid metabolism |
| 7 | - | 6.1414 | 319.2264362 | C20H32O3 | HMDB0002232 | 8,9-Epoxyeicosatrienoic acid (8,9-EET) | 151,163,165,275,301,319 | 1.10028 | ↓** | C14769 | Arachidonic acid metabolism |
| 8 | - | 6.01625 | 361.23727 | C21H32O2 | HMDB0000253 | Pregnenolone | 271,279,287,297,299 | 1.20183 | ↑** | C01953 | Steroid hormone biosynthesis |
| 9 | - | 6.005533333 | 337.2346084 | C20H34O4 | HMDB0002995 | 12-Keto-tetrahydro-leukotriene B4 | 293,319,337 | 1.19968 | ↑** | C02165 | Arachidonic acid metabolism |
| 10 | - | 5.083766667 | 333.2060696 | C19H28O2 | HMDB0000899 | Androstanedione | 83,100,145,151,161,163,165,169,175,177,215,217,223,225,227, 240,25,265,269,285 | 1.08321 | ↓** | C00674 | Steroid hormone biosynthesis |
| 11 | - | 4.98005 | 437.2884159 | C24H40O4 | HMDB0000518 | Chenodeoxycholic acid | 311,314,325,343,353,366,371,373,391 | 1.11693 | ↓** | C02528 | Primary bile acid biosynthesis |
| 12 | - | 4.594233333 | 335.2215555 | C7H15NO2 | HMDB0001161 | 4-Trimethylammoniobutanoic acid | 58,85,99,114,144 | 1.07781 | ↑** | C01181 | Lysine degradation |
| 13 | - | 3.879533333 | 453.2828898 | C24H40O5 | HMDB0000432 | 3α,7β,12α-Trihydroxy-5α-Cholanoic acid | 345,371,377,389,407 | 1.15873 | ↓** | C02373 | Steroid hormone biosynthesis |
| 14 | - | 3.797383333 | 335.221456 | C20H32O4 | HMDB0001193 | 5(S)-Hydroperoxyeicosatetraenoic acid  (5(S)-HPETE) | 89,115,259,273,303,317 | 1.19252 | ↑** | C05356 | Arachidonic acid metabolism |
| 15 | - | 3.543616667 | 997.5865802 | C26H45NO6S | HMDB0000951 | Taurochenodesoxycholic acid | 304,391,433,480.481,498,499 | 1.20008 | ↑** | C05465 | Primary bile acid biosynthesis |
| 16 | - | 3.161383333 | 244.9985913 | C5H12O7P2 | HMDB0001120 | Dimethylallylpyrophosphate | 158,182,200,226 | 1.20288 | ↑** | C00235 | Terpenoid backbone biosynthesis |
| 17 | - | 3.072083333 | 355.0716826 | C5H10N2O3S | HMDB0000078 | Cysteinylglycine | 97,99,100,102,113,117,126,131,133,177 | 1.1937 | ↓** | C01419 | Glutathione metabolism |
| 18 | - | 2.98265 | 197.0443 | C8H8O3 | HMDB0003791 | 3,4-Dihydroxyphenylacetaldehyde | 121,123,133,135,151 | 1.15774 | ↓** | C04043 | Tyrosine metabolism |
| 19 | - | 2.428833333 | 209.0782993 | C3H7NO3 | HMDB0003406 | D-Serine | 56,58,72,86,104 | 1.20741 | ↑** | C00740 | Glycine, serine and threonine metabolism; D-Amino acid metabolism |
| 20 | - | 2.1323 | 195.062535 | C2H5NO2 | HMDB0000123 | Glycine | 74 | 1.20325 | ↑** | C00037 | Glycine, serine and threonine metabolism;Glyoxylate and dicarboxylate metabolism ;Porphyrin metabolism; Glutathione metabolism; Lipoic acid metabolism |
| 21 | - | 1.413916667 | 88.03951238 | C3H7NO2 | HMDB0000161 | L-Alanine | 88 | 1.19284 | ↑** | C00041 | Selenocompound metabolism; Alanine, aspartate and glutamate metabolism |
| 22 | - | 1.392483333 | 225.0868724 | C9H12N2O2 | HMDB0004076 | 5-Hydroxykynurenamine | 179,162,161 | 1.1917 | ↑** | C05638 | Tryptophan metabolism |
| 23 | - | 1.17815 | 291.039057 | C5H6O5 | HMDB0000208 | Oxoglutaric acid | 73,101,144 | 1.05764 | ↓** | C00026 | Butanoate metabolism; Lipoic acid metabolism; Alanine, aspartate and glutamate metabolism; Citrate cycle (TCA cycle); Citrate cycle (TCA cycle) |
| 24 | - | 1.174566667 | 103.0389122 | C4H8O3 | HMDB0000011 | 3-Hydroxybutyric acid | 59,102 | 1.07608 | ↓** | C01089 | Butanoate metabolism |
| 25 | + | 10.70118333 | 1147.817094 | C33H54O7 | HMDB0010330 | Cholesterol glucuronide | 111,273,327,369,563 | 2.089 | ↓** | C03033 | Pentose and glucuronate interconversions |
| 26 | + | 10.68688333 | 923.6627704 | 10.68688333 | HMDB0000601 | Coprocholic acid | 317,330,331,369,421,451 | 2.4493 | ↓** | C04722 | Primary bile acid biosynthesis |
| 27 | + | 9.884466667 | 828.5483613 | C42H79NO13 | HMDB0004866 | LacCer(d18:1/12:0) | 628,788 | 5.06864 | ↓** | C01290 | Sphingolipid metabolism |
| 28 | + | 5.543016667 | 400.3404595 | C23H45NO4 | HMDB0000222 | Palmitoylcarnitine | 341, 400 | 4.83757 | ↓** | C02990 | Fatty acid degradation |
| 29 | + | 2.556316667 | 339.0172494 | C3H7NO5S | HMDB0002757 | Cysteic acid | 105,124,137,170 | 1.77255 | ↓** | C00506 | Cysteine and methionine metabolism; Taurine and hypotaurine metabolism |
| 30 | + | 1.125766667 | 182.0806595 | C9H11NO3 | HMDB0000158 | L-Tyrosine | 94,106 | 1.76917 | ↓** | C00082 | Tyrosine metabolism; Phenylalanine, tyrosine and tryptophan biosynthesis; Ubiquinone and other terpenoid-quinone biosynthesis; Phenylalanine metabolism |
| 31 | - | 3.282833333 | 464.2991571 | C26H43NO6 | HMDB0000138 | Glycocholic acid | 464 | 1.79885 | ↓** | C01921 | Primary bile acid biosynthesis |
